# Identification and Validation of Hypoxia-Related lncRNA Signature as a Prognostic Model for Hepatocellular Carcinoma

**DOI:** 10.3389/fgene.2021.744113

**Published:** 2021-09-28

**Authors:** Chenghui Zhou, Huajun Zhang, Liqing Lu

**Affiliations:** ^1^ Department of General Surgery, Xiangya Hospital, Central South University, Changsha, China; ^2^ Department of General, Visceral, Cancer and Transplantation Surgery, University Hospital Cologne, Cologne, Germany; ^3^ Key Laboratory of Cancer Proteomics of Chinese Ministry of Health, Xiangya Hospital, Central South University, Changsha, China

**Keywords:** hepatocellular carcinoma, hypoxia, lncRNA, prognosis, immune cells infiltration, immune checkpoint, m6A

## Abstract

Hepatocellular carcinoma (HCC) is one of the most general malignant tumors. Hypoxia is a critical clinical characteristic and acts as a significant part in the development and cancers’ prognosis. The prognostic value and biological functions of hypoxia-related lncRNAs in hepatocellular carcinoma is little known. Thus, we aim to establish a hypoxia-related lncRNA signature to predict the HCC patients’ survival. First, we extracted the hypoxia-related genes and expression of lncRNAs from the MSigDB and TCGA database, respectively. The co-expression analysis among hypoxia-related mRNAs and lncRNAs was employed to identify hypoxia-related lncRNAs. Then, comprehensive analyses of lncRNAs expression level and survival data were applied to establish the signature. We built a prognostic signature on the foundation of the three differently expressed hypoxia-related lncRNAs. Kaplan-Meier curves indicated the low-risk group is associated with better survival. The 1−, 3−, and 5 years AUC values of the signature were 0.805, 0.672 and 0.63 respectively. The test set performed consistent outcomes. A nomogram was built grounded on the risk score and clinicopathological features. GSEA showed the immune-related pathways in high-risk group, while metabolism-related pathways in low-risk group. Besides, we found this model was correlated with the clinical features, tumor immune cell infiltration, immune checkpoints, and m6A-related genes. Finally, a novel signature based on hypoxia-related lncRNAs was established and validated for predicting HCC patients’ survival and may offer some useful information for immunotherapies.

## Introduction

Hepatocellular carcinoma (HCC) one of the most general subtype of liver cancer, ranks the fifth in terms of cancer-related deaths in the world (Bray et al., 2018). The surgical treatment, radiofrequency ablation, chemotherapy and as well liver transplant are the traditional treatments for hepatocellular carcinoma patients and the therapeutic effects are limited. The majority of patients are diagnosed with advanced stage and there are distant metastasis, tumor thrombosis of portal vein, and so on, leading to extremely poor prognosis (Galle et al., 2017; Vilgrain et al., 2017). Nowadays, in China, the survival rate of 5 years of HCC patients is only 14.1% (Allemani et al., 2018). Thus, novel predictive biomarkers and therapeutic options are critically required.

Hypoxia belongs to a public microenvironmental property of most solid tumors, because of the imbalance between the tumor proliferation and oxygen supply (Hill et al., 2015; Muz et al., 2015). Previous studies have proved that hypoxia plays critical roles in angiogenesis, differentiation, proliferation and apoptosis of tumor cells (Nishida and Kudo, 2013; Wu et al., 2007). As one of the organs most prone to hypoxia, hypoxia is correlated with the metastasis, prognosis, and resistance of HCC (Erler and Giaccia, 2006; Graham and Unger, 2018). Whereas lncRNA is a subset of RNA transcripts over 200 nt, which not only regulates the mRNA’s expression but also mediates the development, occurrence, metastasis and prognosis of various cancers (Kang et al., 2019; Gao et al., 2021; Xiao et al., 2021). At present, multiple studies found that hypoxia-related lncRNAs are associated with overall survival in various tumors (Chen et al., 2020; Zhang et al., 2021). Nonetheless, whether the hypoxia-related lncRNAs are related to the prognosis of hepatocellular carcinoma is still largely not clear.

Herein, we mined the expression data of lncRNAs and the relevant clinical information of hepatocellular carcinoma samples, and hypoxia-related genes from the opening databases. Next, the prognostic signature of hypoxia-related lncRNA was built and then evaluated it based on the TCGA cohort. Lastly, we validated the predictive capability of the risk model and explored the role in diagnostic value, infiltrating immune cells, N6-methyladenosine (m6A) mRNA, and immune checkpoints.

## Materials and Methods

### Data Collection

The RNA-sequencing (FPKM) of 374 hepatocellular carcinoma samples was derived from the TCGA data portal. The corresponding data of clinical, for example, age, gender, clinical grade, clinical stage and survival information, was also downloaded from the TCGA database. The clinical information of these patients is displayed in Table 1. The Ensembl was applied to obtain the gene transfer annotation files to differentiate the messenger RNAs (mRNAs) from long non-coding lncRNAs (lncRNAs) for subsequent analyses. Besides, the merge language script Perl was used to merge the data files of RNA-sequencing into a matrix file. Then, the genes’ Ensembl IDs were translated into an array of gene symbols by utilizing the script of the Perl language.

**TABLE 1:** The clinical information of hepatocellular carcinoma patients in the whole cohort.

Variable	Number of patients
**Age at diagnosis**	
≤65	216
>65	127
**Gender**	
Female	110
Male	233
**Grade**	
G1	53
G2	161
G3	112
G4	12
Unknown	5
**Stage**	
I	161
II	77
III	80
IV	3
Unknown	22

Additionally, the Molecular Signatures database (MSigDB) (M10508, M26925) was applied to download the hypoxia-related genes. The co-expression analysis was employed to identify hypoxia-related lncRNAs. The hypoxia-related genes with correlation coefficients > 0.5 and *p*-value < 0.001 were regarded as hypoxia-related lncRNAs. The lncRNAs with the average expression less than 0.5 were delated. The expression profiles of hypoxia-related genes/lncRNAs of the 50 adjacent normal tissue samples and 374 samples of hepatocellular carcinoma were subjected to DE-HRGs/DE-HRlncRNAs using the “limma” package in R. The GO and KEGG were applied to explore the potential function of the differentially expressed hypoxia-related genes (DE-HRGs) (FDR < 0.05 and |log2FC| ≥ 1) using the “clusterProfiler”, “enrichplot” and “ggplot2” R packages. The samples of hepatocellular carcinoma were distributed into the train and test sets applying the “caret” package randomly. We built the prognostic signature based on the hypoxia-related lncRNAs’ expression level in the train set and validated its prognostic value in the test set.

### Construction of a Hypoxia-Related lncRNA Signature to Assess the Risk Score

To identify the hypoxia-related lncRNAs that related overall survival, we merged the hypoxia-related lncRNAs and survival information utilizing the package “limma”. The survival time of hepatocellular carcinoma samples ranks from 0 to 3,675 days. To improve the quality analyses, samples that lack complete survival information and those whose overall survival was not over 30 days were excluded from this analysis. Then, the univariate Cox regression analysis was applied to get survival-related hypoxia-related lncRNAs from the hypoxia-related lncRNAs (*p* < 0.05). These survival-related hypoxia-related lncRNAs were used for further analysis.

The LASSO Cox analysis was employed to identify the prognostic hypoxia-related lncRNAs. To prevent overfitting, we applied 1000-round cross-validation for tuning parameter selection to fulfill the minimum criteria of the partial likelihood deviance. Finally, the multivariate Cox regression analysis was fulfilled on these prognostic hypoxia-related lncRNAs to build a prognostic hypoxia-related lncRNAs signature and compute the coefficients. The risk score for each sample was counted utilizing the following formula: risk score = ∑icoefficient (hypoxia-related lncRNA) × expression of (hypoxia-related lncRNAi).

### Validation of the Hypoxia-Related lncRNA Signature

The patients of train and test sets were divided into two subgroups (low-risk vs high-risk) on the foundation of the median value of the risk score of train cohort, respectively. We applied Kaplan-Meier survival analysis to explore the difference of survival between the two groups in the train and test sets. The time-dependent receiver operating characteristic (ROC) curves of 1-, 3- and 5 years were plotted using the “survivalROC” package in two sets. Besides, to explore whether the risk score can regard as an independent biomarker for the hepatocellular carcinoma patients’ survival, the univariate and multivariate Cox regression analysis were used in the train and test sets. To prove the clinical application value of this signature, we applied the chi-square test to explore the relationship between the model and clinical variables. We compared the differences in the risk score among subgroups with different clinical features with a Wilcoxon signed-rank test.

### Construction of Nomogram and Gene Set Enrichment Analysis

A nomogram was built on the foundation of the risk score and other clinical biomarkers for predicting the 1-, 3-, 5 years overall survival of hepatocellular carcinoma patients. We applied the calibration curves to assess the nomogram’ accuracy. The Gene set enrichment analyses (GSEA) was performed to investigate the KEGG pathways between two risk (low vs high) groups in whole cohort of TCGA-LIHC using GSEA 4.0.1 software. *p* < 0.05 and FDR < 5% were considered as statistical significance.

### Association Between Risk Scores and Immune Cell Infiltration

To explore the association between the risk score and features of immune cell infiltration, we used the various acknowledged methods to count the tumor infiltrating immune cells levels among the patients from the whole cohort, including CIBERSORT-ABS, XCELL, EPIC, MCPcounter, TIMER, QUANTISEQ, and CIBERSORT. We used the Wilcoxon signed-rank test to explore the difference in immune cell infiltration level between two risk groups. To explore the association between two risk groups and immune status, we quantified the tumor-infiltrating cells related pathways with ssGSEA (Xiao et al., 2020), namely, APC_co_inhibition, APC_co_stimulation, CCR, check-point, cytolytic activity, HLA, inflammation promoting, MHC_class_I, parainflammation T cell co-inhibition, T cell co-stimulation, type I_IFN response, and type II_IFN response. Previous studies have proved that m6a-related genes were associated with prognosis in hepatocellular carcinoma (Zhao et al., 2018; Wu et al., 2020). What is more, m6a-related genes can mediate the expression of lncRNAs, which dyregulate the tumorigenesis, development, metastasis, as well as prognosis (Lan et al., 2019; Wu et al., 2021a). Thus, we evaluated the association between the risk score and N6-methyladenosine (m6A) mRNAs as well as immune checkpoints in the whole cohort. The m6A mRNAs and immune checkpoints were obtained from previous literature.

## Results

### Enrichment Analysis of Hypoxia-Related Genes

**FIGURE 1:**
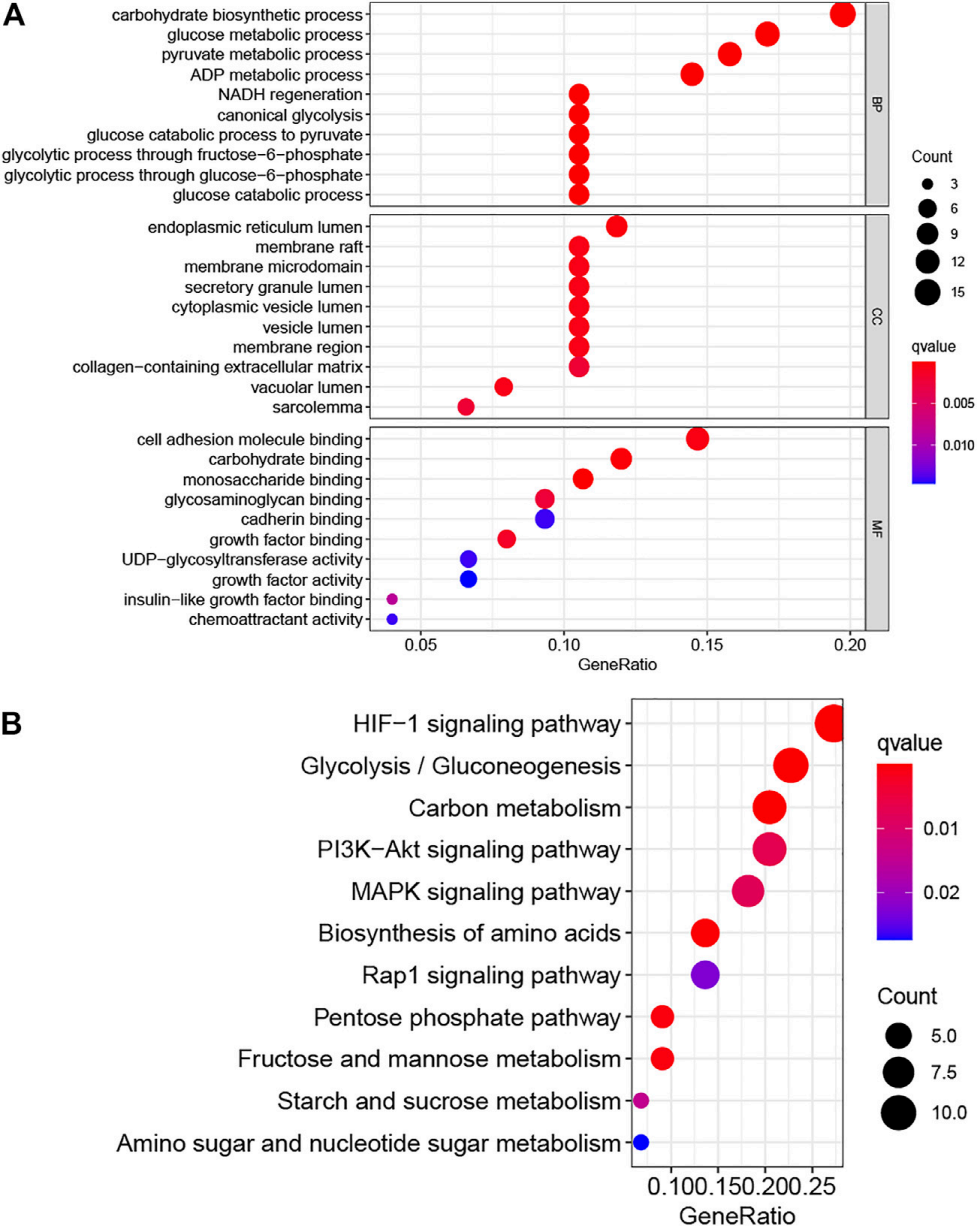
GO **(A)** and KEGG **(B)** analysis for hypoxia-related differentially expressed genes.

We obtained 76 DE-HRGs including 58 upregulated and 18 downregulated genes (Supplementary Table S1). The most enriched GO terms were CC (cellular component) such as membrane raft, endoplasmic reticulum lumen, secretory granule lumen, membrane microdomain and cytoplasmic vesicle lumen; BP (biological process), for example, glucose catabolic process to pyruvate, NADH regeneration, carbohydrate biosynthetic process, glucose metabolic process and canonical glycolysis; and MF (molecular function) such as glycosaminoglycan binding, monosaccharide binding, carbohydrate binding, growth factor binding, and cell adhesion molecule binding (Figure 1A, Supplementary Table S2). The KEGG pathways of 76 DE-HRGs were predominantly related to glycolysis/gluconeogenesis, starch and sucrose metabolism, fructose and mannose metabolism, as well as pentose phosphate pathway (Figure 1B, Supplementary Table S2).

### Establishment of Hypoxia-Related lncRNA Signature

We obtained 200 hypoxia-related genes from the MSigDB database (Supplementary Table S3). Then, a total of 171 hypoxia-related lncRNAs were identified by applying co-expression analysis between hypoxia-related genes and lncRNAs. Among these hypoxia-related lncRNAs, 111 lncRNAs were differentially expressed hypoxia-related lncRNAs (Supplementary Figures S1A,B). Then, we added the hypoxia-related lncRNAs with survival data by merging complete survival material of samples and hypoxia-related lncRNAs. Next, 343 hepatocellular carcinoma samples were distributed into the train (243) and test (100) sets applying the “caret“ package randomly. The samples in the train set were employed for constructing the prognostic model.

**FIGURE 2:**
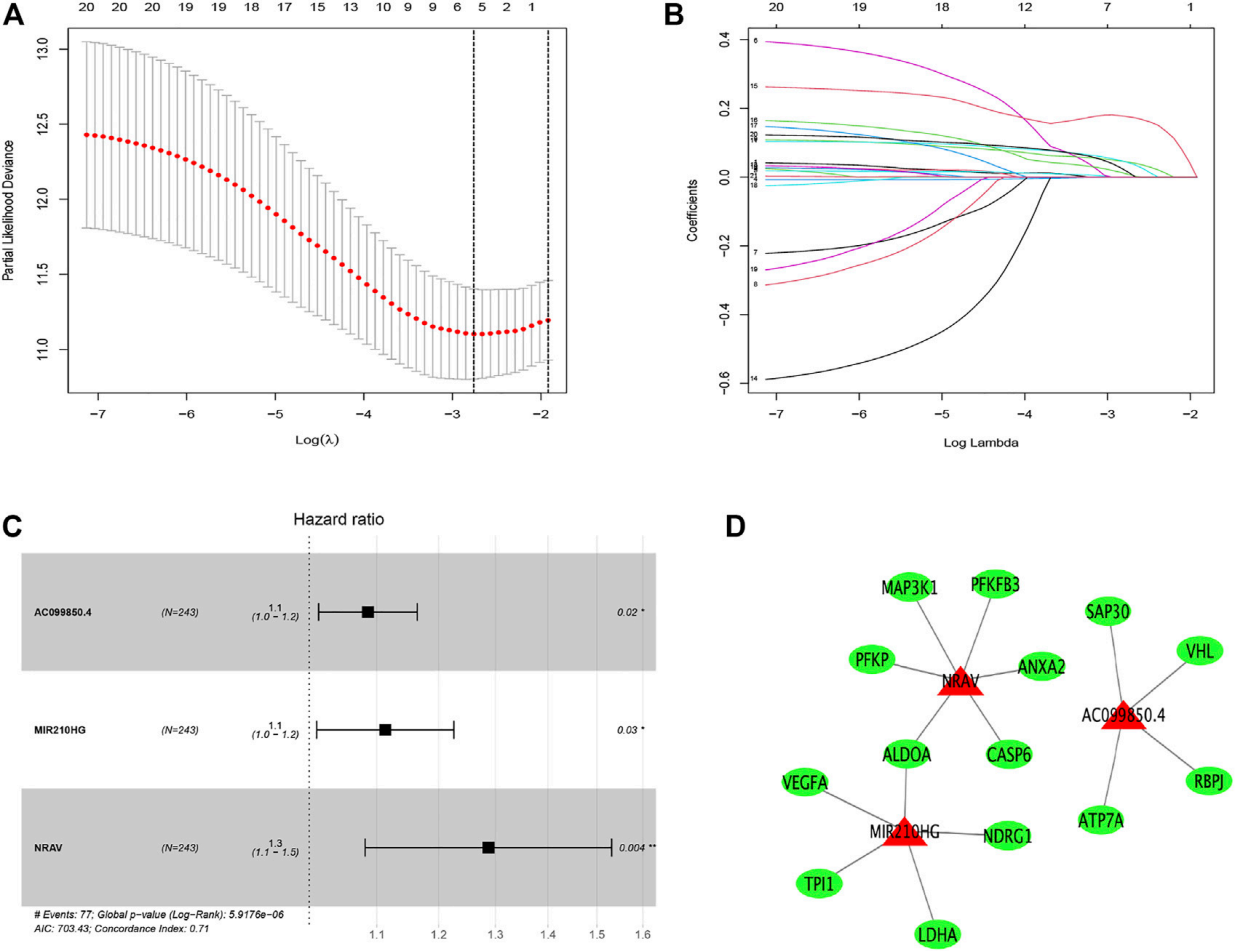
Distinction of hypoxia-related lncRNAs signature: LASSO **(A,B)** and multivariate **(C)** Cox regression analysis. The relationship between the prognostic lncRNAs (red) and genes (green) **(D)**.

First, we identified 21 prognostic hypoxia-related lncRNAs utilizing univariate Cox regression analysis, as shown in Supplementary Table S4. Then, utilizing LASSO regression analysis, 16 hypoxia-related lncRNAs were removed (Figures 2A,B). Finally, three hypoxia-related lncRNAs (AC099850.4, MIR210HG, and NRAV) were obtained through using multivariate Cox regression analysis (Figure 2C). The hypoxia-related lncRNAs signature was constructed based on these three lncRNAs and corresponding coefficients: risk score = (0.08283797* expression of AC099850.4) + (0.10722211* expression of MIR210HG) + (0.25250738* expression of NRAV). Besides, the association between three hypoxia-related lncRNAs in the signature and hypoxia-related genes is displayed in Figure 2D.

### Assessment of Clinical Performance of Risk Model

**FIGURE 3:**
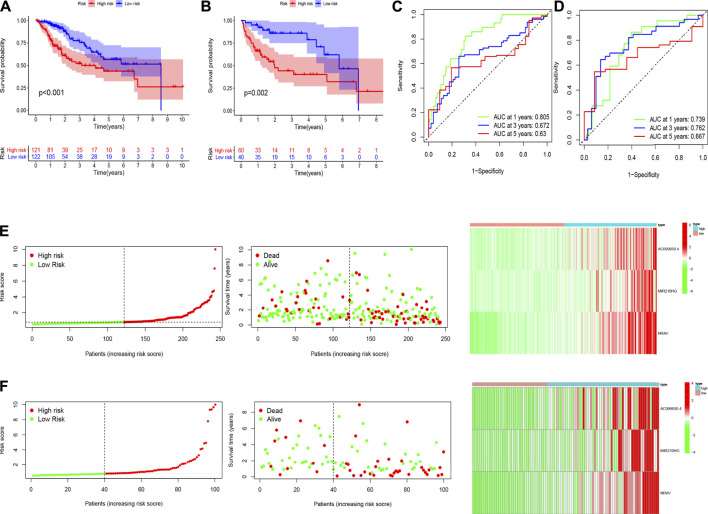
The results of Kaplan-Meier analysis, ROC curves, and risk survival status plots in train **(A,C,E)** and test **(B,D,F)** sets.

To explore the underlying prognostic capability of the hypoxia-related lncRNA signature, we assigned the patients in train and test sets into two groups (low-risk vs high-risk) on the foundation of the median of risk score. The Kaplan-Meier analysis demonstrated the high-risk group was correlated with worse survival (*p* < 0.001, Figure 3A) in the train set and the K-M analysis in the test set revealed the similar result (*p* = 0.002, Figure 3B). Meanwhile, the 1-, 3-, and 5 years AUC values of ROC curve were 0.805, 0.672 and 0.63, respectively, in the train set (Figure 3C). The corresponding values in the test set were 0.739, 0.762 and 0.667, respectively (Figure 3D). Utilizing the risk survival status plots of patients, we revealed that the risk scores of patients were negatively correlated with the hepatocellular carcinoma patients’ survival in train and test sets. Besides, the heatmaps showed that these three lncRNAs were positively associated with the risk signature in two sets (Figures 3E,F).

**FIGURE 4:**
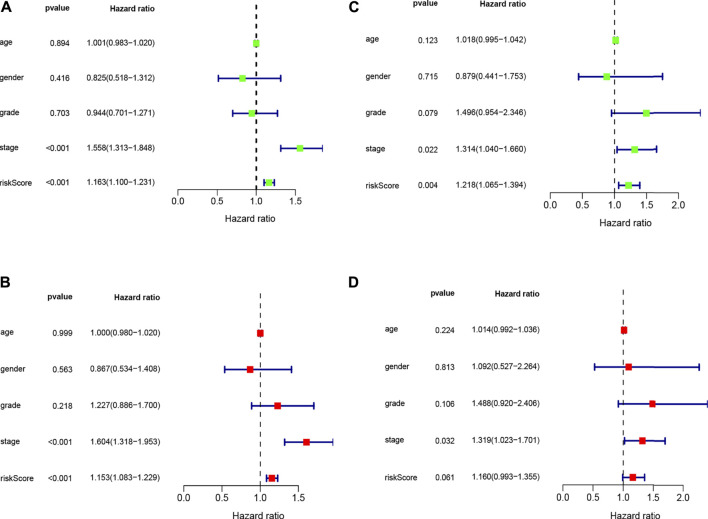
Univariate **(A,C)** and multivariate **(B,D)** COX analysis for risk score and clinical variables in train and test sets.

**FIGURE 5:**
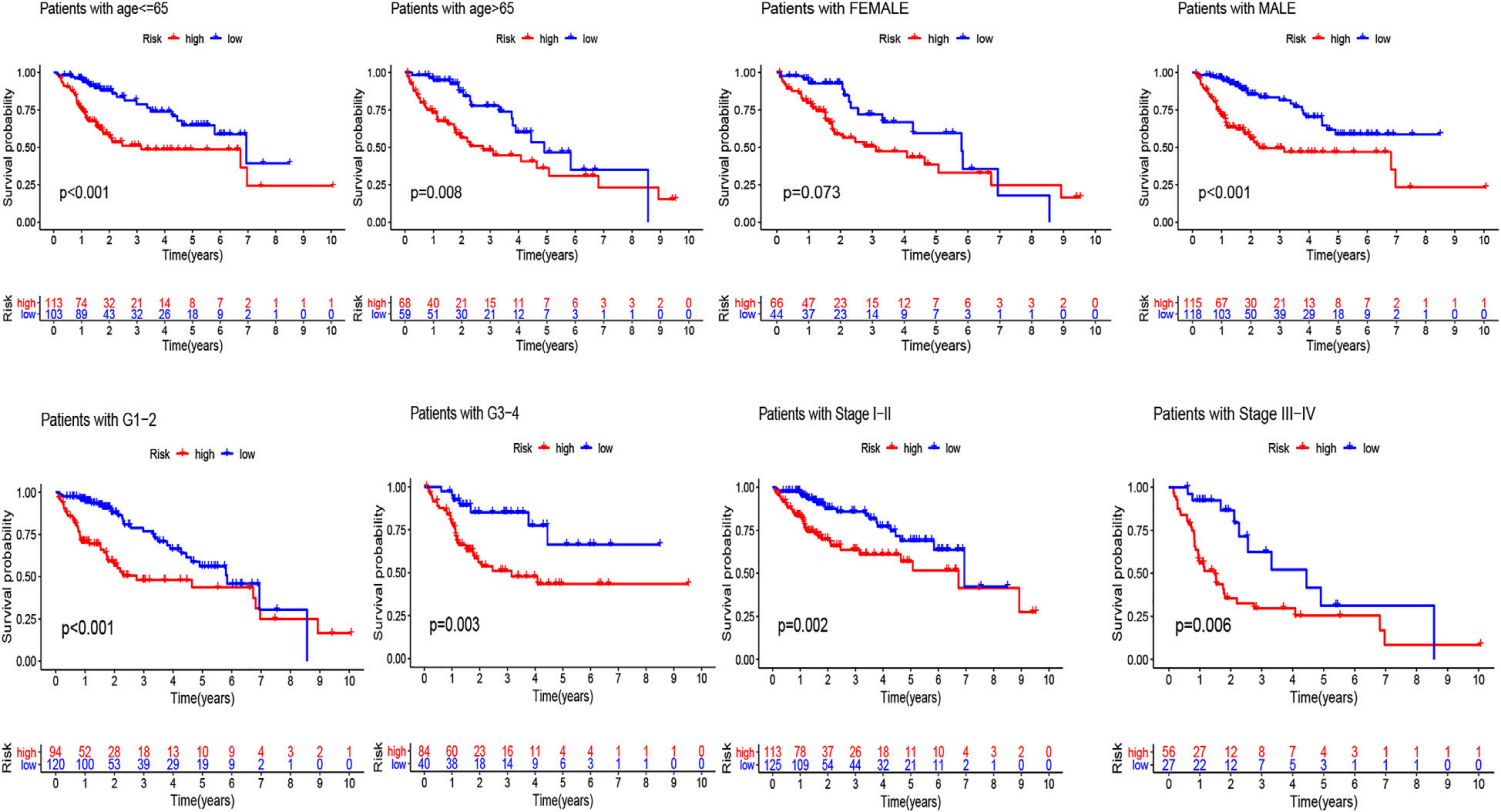
Kaplan-Meier analyses for the two subgroups (high-risk vs low-risk) categorized by clinical variables, comprising age, gender, grade, and stage.

The univariate Cox regression analysis revealed that risk score (HR = 1.163, 95% CI: 1.100–1.231, *p* < 0.001) and stage (HR = 1.558, 95% CI: 1.313–1.848, *p* < 0.001) were remarkably associated with overall survival in the train set (Figure 4A), and risk score (HR = 1.218, 95% CI: 1.065–1.394, *p* = 0.004) and stage (HR = 1.314, 95% CI: 1.040–1.660, *p* = 0.022) were also associated with overall survival in the test set (Figure 4C). Furthermore, the multivariate Cox regression analysis also demonstrated that risk score (HR = 1.153, 95% CI: 1.318–1.953, *p* < 0.001) and stage (HR = 1.604, 95% CI: 1.313–1.848, *p* < 0.001) were the independent factors to predict the hepatocellular carcinoma patients’ prognosis in the train set (Figure 4B), and risk score (HR = 1.160, 95% CI: 0.993–1.355, *p* = 0.061) and stage (HR = 1.319, 95% CI: 1.023–1.701, *p* = 0.032) were also the independent factors in the test set (Figure 4D). Subsequently, we applied the ROC curve to appraise the accurateness of clinical variables in predicting the HCC patients’ overall survival. As shown in Supplementary Figure S1C, the AUC value of clinical variables was lower than that of risk score. Besides, to further explore the signature’s prognostic value in hepatocellular carcinoma patients arranged by clinical variables, the patients were divided into distinct subgroups according to clinical variables (age, sex, stage and clinical grade). Figure 5 shows that the survival of patients in high-risk group was worse than those in the low-risk group in most of subgroups.

**FIGURE 6:**
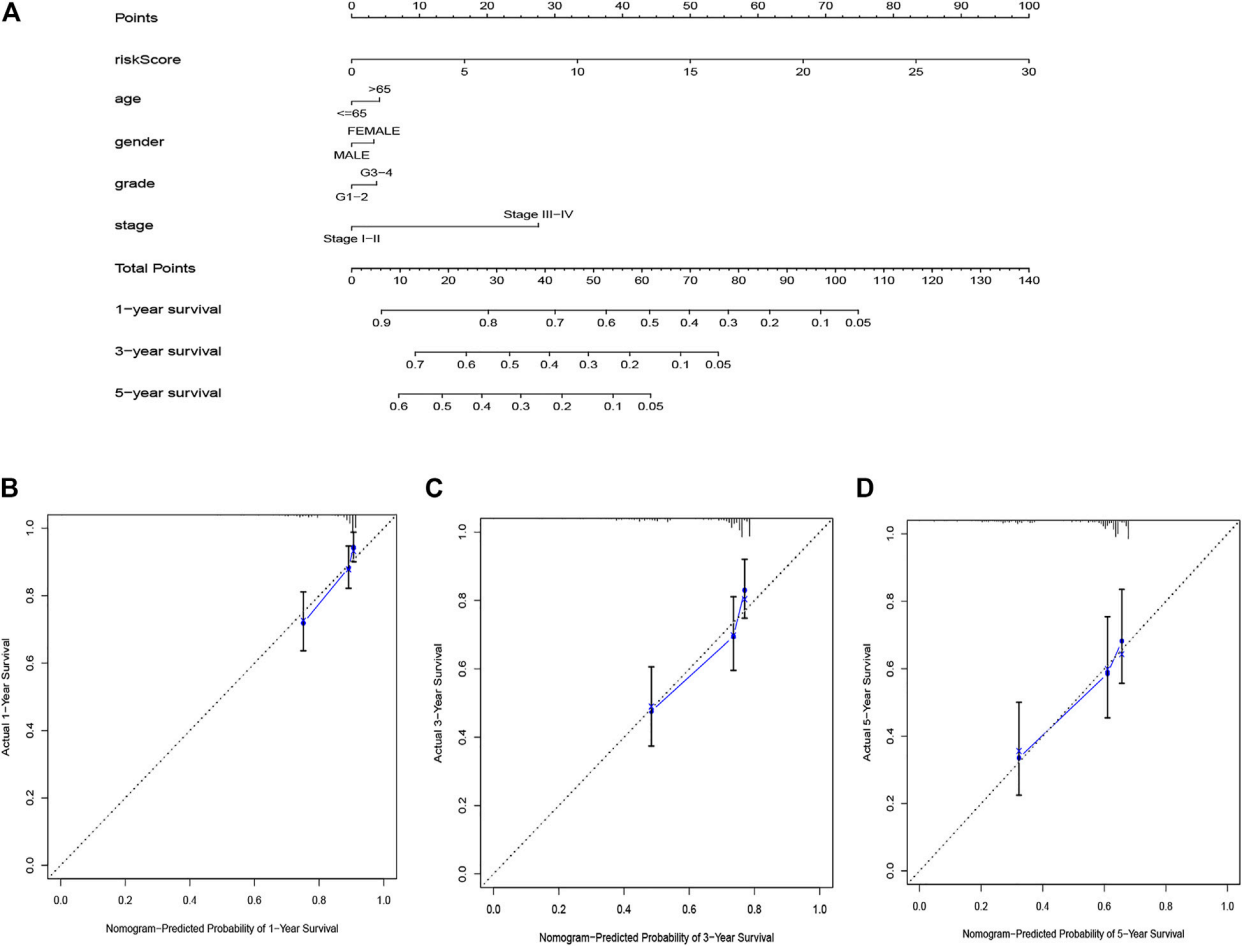
The nomogram for predicting HCC patients’ survival **(A)**. Calibration plots applied for predicting the 1-year **(B)**, 3-year **(C)** and 5-year **(D)** survival.

The nomogram based on the risk score and clinical features was constructed for predicting the 1-, 3- and 5 years overall survival of the hepatocellular carcinoma patients (Figure 6A). The 1-, 3-, and 5 years calibration curves showed stable and accurate performance (Figures 6B–D); therefore, they may be used for hepatocellular carcinoma patients’ clinical management.

### Gene Set Enrichment Analysis

**FIGURE 7:**
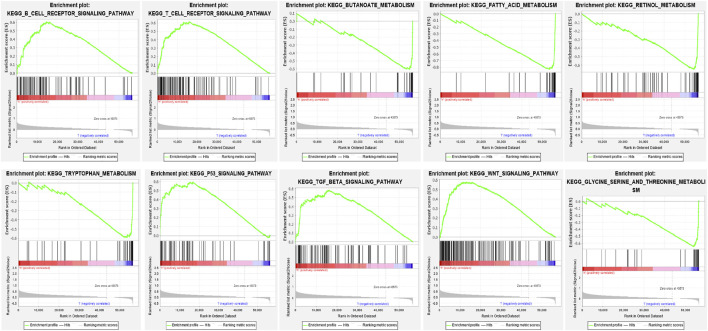
GSEA for two risk groups based on the signature.

GSEA demonstrated the prognostic hypoxia-related lncRNAs signature was enriched in immune-, metabolism- and tumor-related pathways. The high-risk group was enriched in B cell receptor signaling pathway, T cell receptor signaling pathway, P53 signaling pathway, TGF_BETA signaling pathway and WNT signaling pathway, while the low-risk group was enriched in butanoate metabolism, fatty acid metabolism, retinol metabolism, tryptophan metabolism and glycine serine and threonine metabolism (Figure 7).

### Tumor Immune Cell Infiltration and Gene Expression

**FIGURE 8:**
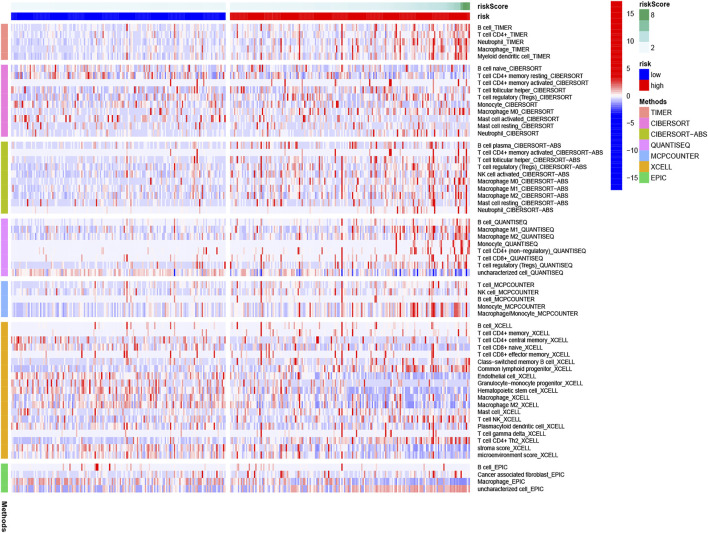
Heatmap for immune cells based on EPIC, CIBERSORT−ABS, CIBERSORT, QUANTISEQ, XCELL, MCP counter, and TIMER algorithms between two risk groups.

**FIGURE 9:**
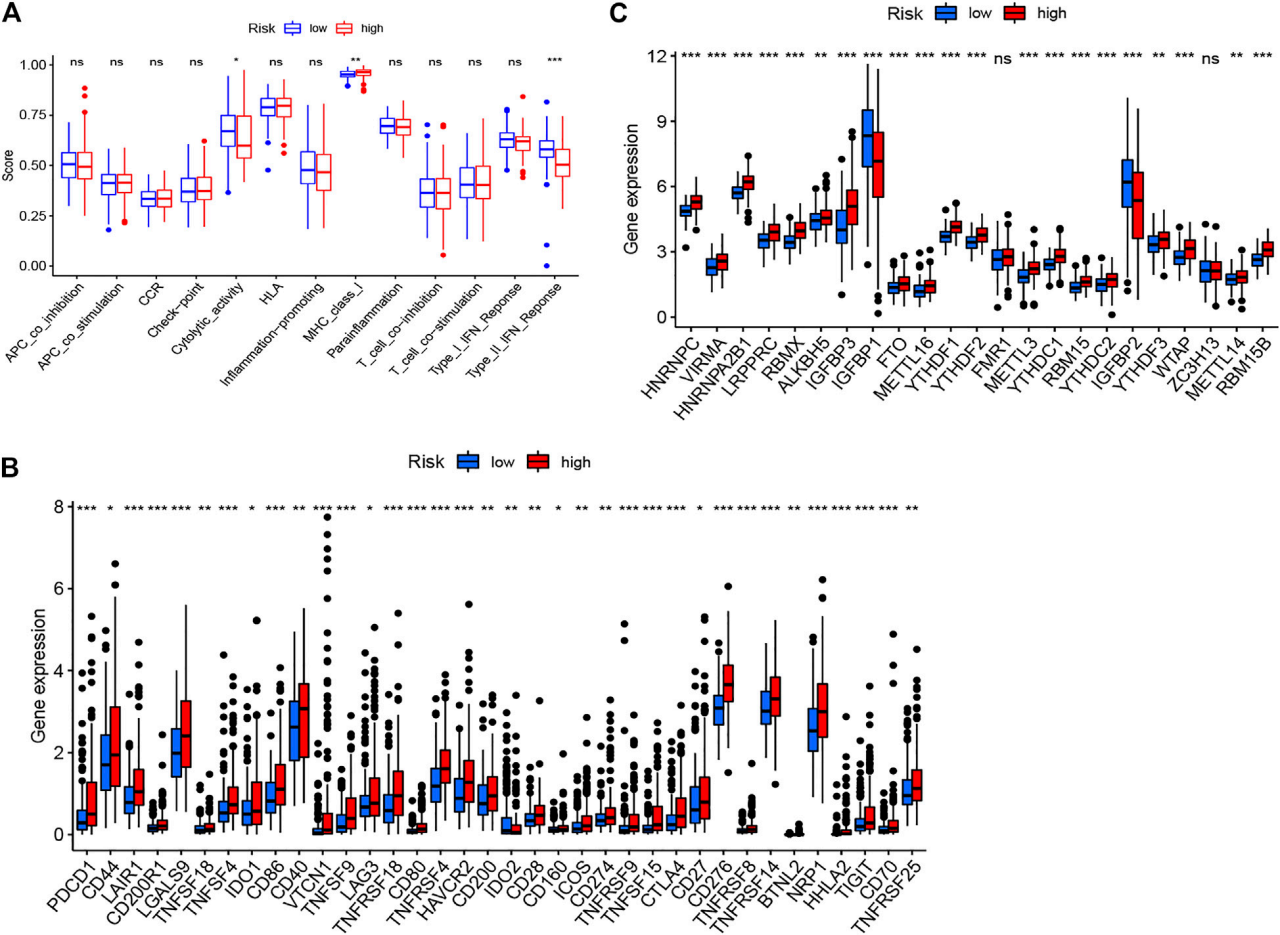
The immune cells related functions **(A)**, immune checkpoints **(B)** and m6A-related genes **(C)** between two risk groups.

The heatmap of tumor immune cell infiltration based on TIMER, EPIC, CIBERSORT−ABS, QUANTISEQ, XCELL, MCP counter and CIBERSORT algorithms is displayed in Figure 8 and Supplementary Table S5. The difference of immune cell related functions indicated that cytolytic activity, type II IFN response and MHC class I were remarkably different between the two risk groups applying the single-sample gene set enrichment analysis (ssGSEA) (Figure 9A). Considering the significance of immune checkpoints in immunotherapies, we next investigated the difference of immune checkpoints’ expression between two risk groups. The boxplot showed most of these genes, for example, PDCD-1 (PD-1), CD86, LAIR1, CTLA4 and CD70, existed significantly different between the two groups (Figure 9B). Besides, most of m6A-related genes’ expression also had significant difference between two risk groups except for FMR1 and ZC3H13 (Figure 9C).

## Discussion

Despite the development of surgical resection, liver transplantation, chemotherapy, immunotherapy, and targeted therapy in the therapy of hepatocellular carcinoma, the hepatocellular carcinoma patients’ prognosis is still poor (Maluccio and Covey, 2012; Xu et al., 2020a). The studies showed that the treatment response and prognosis are influenced by tumor molecular characteristics, although patients own homogeneous clinical risk variables. Thus, it is urgent to identify novel molecular prognostic markers that distinguish from common clinical risk features. Previous studies have proved that hypoxia is associated with tumor angiogenesis, metabolic alteration, metastasis, and prognosis (Krock et al., 2011; Eales et al., 2016; Sun et al., 2020). Meanwhile, lncRNAs play a crucial role in regulating the hypoxia-related genes’ expression. Therefore, we built a signature based on the hypoxia-related lncRNAs and explored tumor immune cell infiltration, immune checkpoints and m6A-related genes in the prognosis of hepatocellular carcinoma, which may offer underlying biomarkers and therapeutic targets for HCC.

First, we explored the function of 76 hypoxia related DEGs. The KEGG analysis indicated these genes mainly enriched in glycolysis/gluconeogenesis, fructose and mannose metabolism, pentose phosphate pathway, as well as starch and sucrose metabolism. Under hypoxia, cancer cells could retain survival and stimulate tumor proliferation, migration, and invasion *via* glycolysis (Huang and Zong, 2017). Wu et al. reported that OMA, an ATP-dependent zinc metalloprotease, promotes cancer development by reprogramming metabolic colorectal cancer under hypoxia (Wu et al., 2021b). A recent study revealed that hypoxia downregulates the enzymes of pentose phosphate pathway and upregulates the enzymes of glycolysis in tumor and then stimulate tumor migration while inhibiting tumor proliferation (Kathagen-Buhmann et al., 2016). Besides, found that hypoxia and the hypoxia inducible factors (HIFs) step in the metabolic crosstalk between tumor cells and their microenvironment (Mucaj et al., 2012). Then, we established a prognostic signature based on three hypoxia-related lncRNAs (AC099850.4, MIR210HG, and NRAV). Wang et al. revealed that high expression of MIR210HG in hepatocellular carcinoma samples and cells not only associated with clinicopathological characteristics, for example, stage, tumor size, vascular invasion, and histological differentiation but also with poor prognosis (Wang et al., 2019). Du et al. revealed MIR210HG is a hypoxia-related lncRNA in triple-negative breast cancer and promotes the Warburg effect by directly regulating HIF-1α (Du et al., 2020). Besides, overexpression of MIR210HG promotes the progression of colorectal adenocarcinoma *via* modulating hypoxia (He et al., 2019) and was associated with worse overall survival in colon cancer patients (Ruan et al., 2019). AC099850.4 is one of the top lncRNA in miRNA–lncRNA network and may be associated with tumor division, tumor proliferation, cell cycle and so on in ovarian cancer (Zhao et al., 2020). Higher expression of NRAV was associated with advanced clinical stage, poor prognosis and immunologic characteristics in hepatocellular carcinoma patients (Feng et al., 2021; Xu et al., 2021). However, to date, there are few studies on these lncRNAs in hepatocellular carcinoma under hypoxia. These findings may offer crucial insights into the cancers’ control in the future.

Herein, we divided the samples into the two risk subgroups on the foundation of the hypoxia-related lncRNA signature to explore the underlying roles in HCC. Hypoxia acts as an important role in anti-tumor immune responses *via* mediating the expression levels of immune checkpoints (Multhoff and Vaupel, 2020). There are very little studies exploring the connection between immune checkpoint inhibitors and hypoxia at present. We compared the difference of immune checkpoints between two risk group and discovered that most of them exist remarkably difference, which may offer underlying therapeutic targets for hepatocellular carcinoma. More and more studies show that miRNA and lncRNA act as an important role in hypoxia. Hypoxia-inducible factor (HIF) is a critical to cellular responses to hypoxic stress. LncRNA SNHG11 promotes invasion and metastasis of colorectal cancer cells by regulating HIF-1α (Xu et al., 2020b). Hypoxia activated lncRNA HABON enhances the proliferation and growth of hepatocarcinoma cells by mediating HIF-1α (Ma et al., 2021). Hypoxia-inducible miR-182 accelerates tumor angiogenesis and growth by regulating the expression level of HIF1α in prostate cancer cells (Li et al., 2015).

Besides, hypoxia can change the tumor cells’ interplay and crosstalk with the tumor micro-environment, resulting to immune suppression and resistance, which contribute tumor cells to escape immune surveillance (Jing et al., 2019; Noman et al., 2019). To explore the relationship between our signature and tumor micro-environment, we fulfilled GSEA. The outcomes revealed that various immune-related pathways participated in the high-risk group, while the metabolism-related pathways participated in the low-risk group. We next plotted the heatmap of immune cell infiltration of HCC patients to explore the difference of immune microenvironment between two risk groups by using multiple algorithms. We found that T cell follicular helper, NK cell, macrophage M0, macrophages M2, neutrophil, and mast cells resting were highly infiltrated in HCC, associating with tumorigenesis, progression, and metastasis (Zhang et al., 2017; Aponte-López and Muñoz-Cruz, 2020; Ngabire et al., 2020). These results showed that this hypoxia-related lncRNA signature can partially reflect tumor immune cell infiltration and may offer some useful information for immunotherapies. Moreover, majority of m6a-related genes were highly expressed in high risk group, which is similar to previous studies. Chen et al. revealed that WTAP was extremely overexpressed and regarded as an independent prognostic biomarker for HCC patients (Chen et al., 2019). It was reported that FTO was over-expressed in HCC tissue and associated with poor prognosis of HCC patients as well as promoted cell proliferation by mediating the demethylation of PKM2 (Li et al., 2019). YTHDF1 was remarkably up-regulated and positively associated with pathology stage in HCC. K-M curve revealed that higher expression of YTHDF1 is correlated with worse survival (Zhao et al., 2018). What is more, most of immune checkpoints were over-expressed in high risk group, which may provide some underlying targets for immunotherapy. Some of them have proved correlated with poor prognosis in HCC, such as LAG3 (Yarchoan et al., 2017), NRP1 (Lin et al., 2018) and LAIR1 (Wu et al., 2019).

The data of this analysis was downloaded from TCGA database, including complete survival and clinicopathological information of most patients of HCC. Besides, the HCC samples were sufficient enough to divide into train set and test set. Thus, the prognostic signature was constructed and validated using the patients from the single database. However, there were some limitations. First, we did not validate the prognostic model in external database and lack of the validation in clinical practice without enough HCC samples. Second, we did not perform basic experiment to verify the lncRNAs in this study.

## Conclusion

Specific hypoxia associated lncRNAs provide a novel prognostic signature for hepatocellular carcinoma patients and may improve the individualized treatment strategies.

## Data Availability

The datasets presented in this study can be found in online repositories. The names of the repository/repositories and accession number(s) can be found in the article/Supplementary Material.
