# Supplementary material for: Identification and Validation of Hypoxia-Related lncRNA Signature as a Prognostic Model for Hepatocellular Carcinoma
Source: Front Genet. 2021 Sep 28;12:744113. doi: 10.3389/fgene.2021.744113 (PMC8505699; doi:10.3389/fgene.2021.744113)
Supplement: Supplementary file 3 [file Table1.DOCX]

| **Variable** | **Number of patients** |
| --- | --- |
| **Age at diagnosis** |  |
| <=65 | 216 |
| >65 | 127 |
| **Gender** |  |
| Female | 110 |
| Male | 233 |
| **Grade** |  |
| G1 | 53 |
| G2 | 161 |
| G3 | 112 |
| G4 | 12 |
| Unknown | 5 |
| **Stage** |  |
| I | 161 |
| II | 77 |
| III | 80 |
| IV | 3 |
| Unknown | 22 |
