# Supplementary figures and images for: Identification and Validation of Hypoxia-Related lncRNA Signature as a Prognostic Model for Hepatocellular Carcinoma
Source: Front Genet. 2021 Sep 28;12:744113. doi: 10.3389/fgene.2021.744113 (PMC8505699; doi:10.3389/fgene.2021.744113)

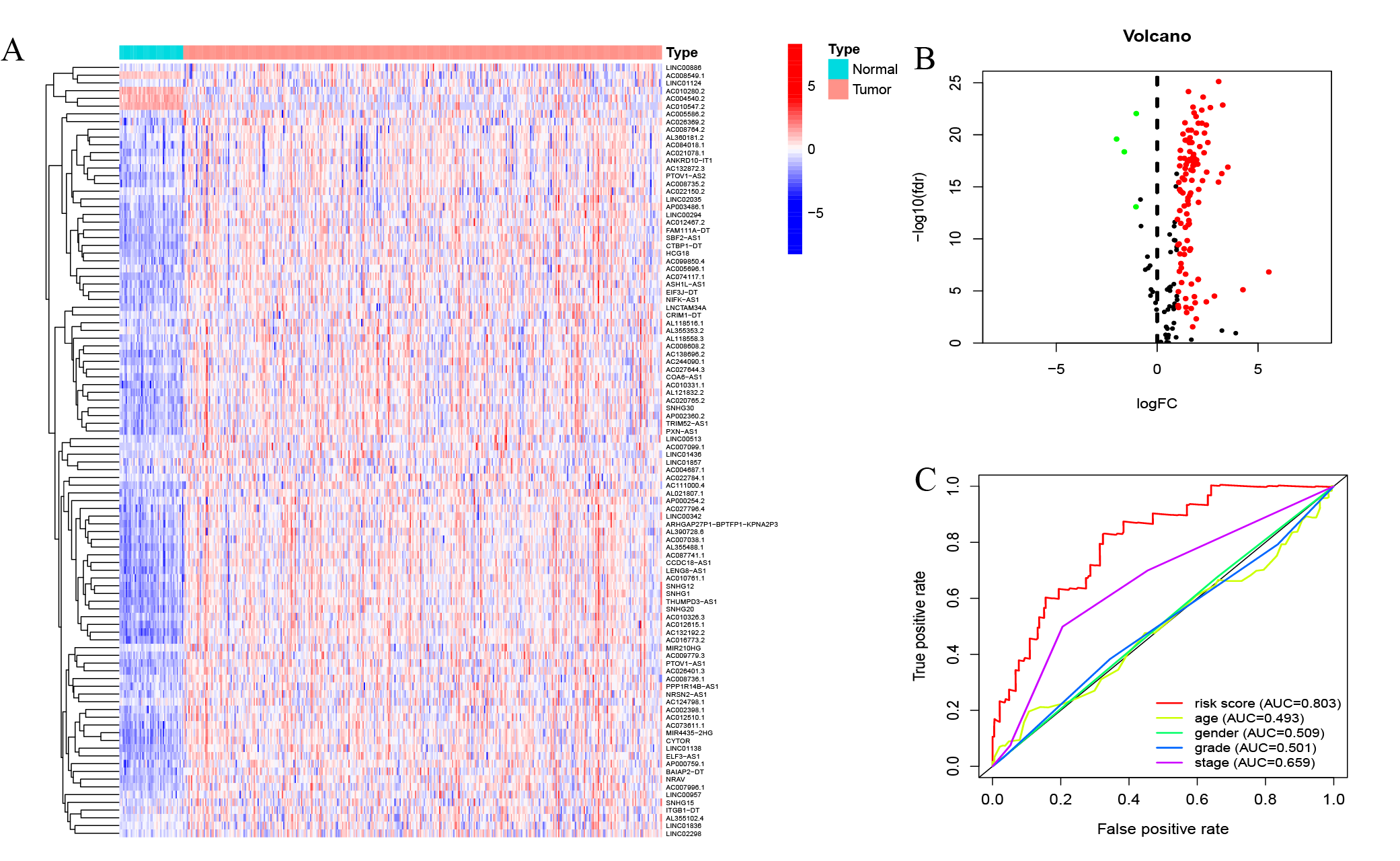

Supplement: Supplementary file 4 [file Image1.TIF]
